# A Neonatal Bimodal MR-CT Head Template

**DOI:** 10.1371/journal.pone.0166112

**Published:** 2017-01-27

**Authors:** Sona Ghadimi, Mehrana Mohtasebi, Hamid Abrishami Moghaddam, Reinhard Grebe, Masoumeh Gity, Fabrice Wallois

**Affiliations:** 1 Faculty of Electrical Engineering, K.N. Toosi University of Technology, Tehran, Iran; 2 Inserm UMR 1105, Faculté de Médecine, Université de Picardie Jules Verne, Amiens, France; 3 Tehran University of Medical Sciences, Tehran, Iran; 4 Inserm UMR 1105, Centre Hospitalier Universitaire d'Amiens, Amiens, France; UNITED STATES

## Abstract

Neonatal MR templates are appropriate for brain structural analysis and spatial normalization. However, they do not provide the essential accurate details of cranial bones and fontanels-sutures. Distinctly, CT images provide the best contrast for bone definition and fontanels-sutures. In this paper, we present, for the first time, an approach to create a fully registered bimodal MR-CT head template for neonates with a gestational age of 39 to 42 weeks. Such a template is essential for structural and functional brain studies, which require precise geometry of the head including cranial bones and fontanels-sutures. Due to the special characteristics of the problem (which requires inter-subject inter-modality registration), a two-step intensity-based registration method is proposed to globally and locally align CT images with an available MR template. By applying groupwise registration, the new neonatal CT template is then created in full alignment with the MR template to build a bimodal MR-CT template. The mutual information value between the CT and the MR template is 1.17 which shows their perfect correspondence in the bimodal template. Moreover, the average mutual information value between normalized images and the CT template proposed in this study is 1.24±0.07. Comparing this value with the one reported in a previously published approach (0.63±0.07) demonstrates the better generalization properties of the new created template and the superiority of the proposed method for the creation of CT template in the standard space provided by MR neonatal head template. The neonatal bimodal MR-CT head template is freely downloadable from https://www.u-picardie.fr/labo/GRAMFC.

## Introduction

Neonatal brain atlases play a crucial role to study normal growth and developmental changes in brain tissues, diagnosis of abnormal anatomical variants, surgical planning, and neuroimaging analysis [1–7]. These atlases are constructed from a population of neurological healthy neonates and represent the typical head structure of healthy neonates.

The majority of published neonatal brain atlases are based on T1- and T2-weighted MR images [7–15]. They are appropriate for brain structural analysis and spatial normalization. Brain atlases include a gray scale anatomical average image (known as template), a set of tissue probability maps (TPMs) and in some cases a set of anatomical parcellation maps These neonatal templates and their associated tissue probability map are used as *a priori* reference to improve segmentation methods [16–19]. However, due to the low intensity of magnetic resonance signals derived from bone, MR templates do not provide the essential accurate details of cranial bones and fontanels-sutures, which are mandatory for the diagnosis of abnormal fontanels [20, 21], a better understanding of the wide variation of normal fontanels and sutures [22] and, more specifically, for functional studies taking into account the impact of fontanels and sutures on neuronal source localization in infants by either high-density (HD) electroencephalography [23–25] or HD functional optical imaging [26]. No appropriate tool is therefore available to segment and create a realistic neonatal skull model from MR images. In a previous study [19], we presented a probabilistic map of the skull based on neonatal MR images. However, this model is not sufficiently accurate and does not provide essential information about the precise location of the fontanels and sutures.

Computed tomography (CT) is the reference modality for skull evaluation, as it provides the best contrast for bone definition. Fontanels and sutures can be distinguished as discontinuities between skull bones. CT images can therefore be used to develop a more realistic model of the neonatal skull. However, because of X-ray exposure, CT acquisition is not recommended for research purposes especially in neonates, but it constitutes the reference modality for clinical assessment of the skull, allowing retrospective research on neonatal skull modeling from normal CT data acquired for clinical purposes in newborns. To the best of our knowledge, only one neonatal CT template is currently available [27], constructed using CT images from 5 subjects using only one image as reference. This template was developed in the framework of a model-based algorithm for skull and fontanel segmentation from CT images.

Since CT images lack sufficient contrast and do not provide useful information for soft tissues, a template based exclusively on CT data only provides information about cranial bones and fontanels-sutures. The CT template, providing information about hard tissues, must therefore be coregistered with the MR template, providing information about soft tissues in the same stereotaxic space. This type of bimodal structural template and associated probability maps for soft tissues and hard tissues would be suitable, after spatial normalization, for the analysis of all structures of the head allowing electrical and optical properties to then be attributed to each precisely segmented structure to facilitate direct and inverse problem in source analysis using EEG or NIRS. Moreover, integration of this type of realistic model of cranial bones based on CT images with the available MR probabilistic maps would provide more accurate results for neonatal MR skull segmentation. In other words, a bimodal MR-CT template may help to construct probabilistic map of cranial bones in the same standard stereotaxic space as introduced by MR template. By using such novel probabilistic map as *a priori* hard tissue information obtained from the CT template and by developing specific algorithms for processing of hard tissue information from MR images, the need for CT acquisition in neonates could be alleviated.

Although a combined MR-CT template is not available for newborns, such a template has been developed in adults stroke patients [28] and has been implemented in SPM toolbox (http://www.mricro.com/clinical-toolbox/spm8-scripts). MR and CT templates are both constructed separately to match the MNI template [29]. Since these adult templates were not directly coregistered with each other, the MR template only approximately matches the shape of the CT template and the accurate shapes of the templates are somewhat different.

Registration of CT images to the neonatal MR template is a crucial step to create a bimodal MR-CT template. This task requires inter-subject inter-modality registration, which is the most challenging aspect of registration, usually used to compare the patient’s data with anatomical atlases. Most of the available methods were developed for adults and intra-subject inter-modality category [30–37]. Typically, pairwise registration is used for generating a template in which a subject image is considered as reference and other images are transferred separately to the space provided by it. This method inevitably causes some bias in the resulted template due to the image selected as reference. Furthermore, since the spatial transformation used for registering the subjects’ image to selected template is estimated independently, it’s not the method of choice in analyzing the group similarity and variation within a population [38–40]. Contrastingly, groupwise registration can simultaneously estimate the transformation fields for all subjects without explicitly specifying an individual subject as reference which provides more accurate and consistent registration among the population [41–45]. By using groupwise registration, the new CT template was then created in full alignment with the MR template. Various published MR templates are available for this age-group [8,11–15], but only two of them [8,13] concern the whole head and contain the skull layer, which is the feature of special interest for the creation of an MR-CT bimodal template. The other MR templates are skull stripped that are not suitable for the creation of the bimodal template. Moreover, these two templates were created nonlinearly, which constitutes an advantage over linearly constructed templates by providing a higher level of detail of anatomical structures of the brain [14].

First, we aim to develop a fully aligned MR-CT bimodal template for neonates with a gestational age (GA) of 39 to 42 weeks. Then, to determine the best approach for inter-subject and inter-modal registration between CT images and MR templates, we develop a two-step intensity-based registration method by globally and locally aligning CT images to the MR template. Finally, we assess the full alignment of our CT template with the corresponding MR one and evaluate it against existing CT templates.

## Materials and Methods

### Subjects and Data Acquisition

In order to create the neonatal brain template, 7 participants (4 girls, 3 boys) were selected from MR images acquired as part of our previously published study [8]. Newborns were imaged with a General Electric 1.5 T MR scanner. The structural 3D volumetric T1-weighted imaging sequence was acquired with the following parameters: TR = 10.1ms, TE = 2.208ms and TI = 500ms. Each 3D volume was constructed from a sequence of slices of 512×512 pixels with 220 mm field of view resulting in a voxel size of 0.47×0.47×0.7mm^3^. Non-axial images were reoriented to the axial plane and all images were re-sliced to 0.47×0.47×0.47mm^3^ isotropic voxels. The neonatal MR template proposed in [8] was used as stereotaxic space to create the CT template.

26 newborn CT images (39–42 weeks GA) were selected from the large database acquired over recent years at Amiens University Hospital, France, many of which have been described in [46]. 16 (12 boys, 4 girls) of these were used to create the CT template and the remaining 10 (5 boys, 5 girls) were used for the cross-validation study. Images were acquired using a LightSpeed 16, GE Medical Systems, with a matrix size of 512×512 pixels and voxel size of [0.26–0.49]×[0.26–0.49]×[0.6–1.25] mm^3^, median 0.32×0.32×0.63 mm^3^. Similar to MR data, all non-axial CT images were reoriented to the axial plane and re-sliced to 0.47×0.47×0.47mm^3^ isotropic voxels. It is worthwhile noting that there was no overlap between the subjects used to create the CT and MR templates.

### CT Template Creation

**Fig 1:**
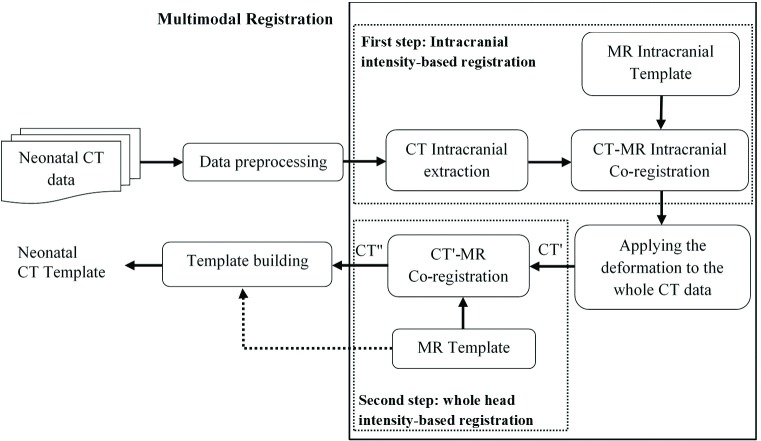
Flow diagram of the proposed pipeline to construct the CT template.

Development of a neonatal bimodal MR-CT template is comprised of two crucial steps: 1) development of an effective two-step intensity-based registration method to align neonatal CT data with the MR template, 2) construction of a neonatal CT template using an advanced groupwise registration and template building method. The selected MR template for this study is the one presented in [8]. Because the only alternative template constructed in [13] presents excessive variations, especially in extracranial parts (http://cmrm.med.jhmi.edu/). The selected template contains the whole neonatal head and matches with the MNI stereotaxic space. An overview of the proposed methodology is shown in Fig 1.

#### Data preprocessing

In order to obtain accurate registration results, CT images were manually rotated and translated in order to be almost aligned with the ‘GRAMFC_T_39-42_’ neonatal MR brain template [8] using 3D Slicer software [47]. During this rigid transformation by 3D Slicer, some voxels with zero intensity may appear in the corners of the image. As typical CT scans were calibrated using Hounsfield units (approximate intensities are: bone: +1000, water: ~0, CSF: ~15, WM: ~20–30, GM: ~37–45, and air: ~-1000), these newly appeared voxels which correspond to background (air voxels) could be wrongly considered as water (CSF) due to their zero intensity. In addition to the aforementioned problem, there are noisy patterns around the head (especially posteriorly due to the pillow and mattress), inappropriate objects such as a pacifier, anesthesia mask or a nasogastric or orogastric feeding tube, and an artificial rim around the air with an intensity of -3000 Hounsfield units (HU) in CT images. These irrelevant structures must be eliminated from CT scans due to their unwanted effect on registration. For this purpose, the head mask of each CT image was extracted using histogram thresholding by Otsu’s method [48] and morphology operators such as opening, closing, filling and identifying connected components. All voxels outside the head mask of each image were then considered to be air and were assigned an intensity value of -1000 HU.

**Fig 2:**
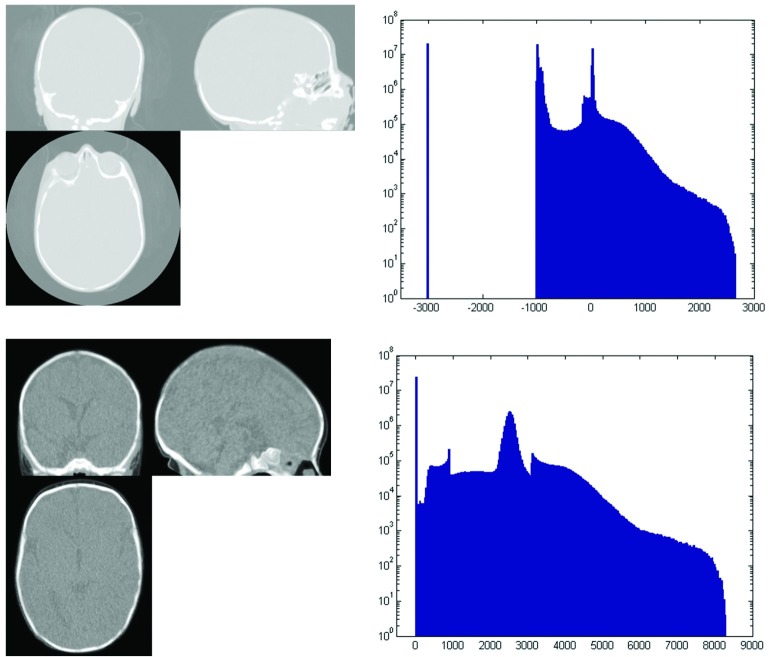
Original and preprocessed CT images and related histogram representation.

CT and MR images are very different in terms of intensity range. CT images typically have an intensity range from -1024 to 2048 and MR images have an intensity range from 0 to 1023 (255 for 8-bit MR images). Ideally, for registration purposes, the intensity value of CT image voxels should be mapped so that the intensity range is similar to the MR intensity range. In particular, it is important to map negative CT values to the non-negative range and expand the dynamic range of soft tissue voxels inside the cranial bones (as in MR images) [28, 36, 49–50]. This type of mapping would allow more accurate differentiation of intracranial soft tissues that present low contrast on raw brain CT images. According to [28], CT image intensities are transformed as follows: values -1000 to -100 are transformed to 0–900, values from -99 to 100 are linearly scaled to the range of 901 to 3100 and intensities higher than 100 (I > 100) are converted to I + 3000 (Fig 2). This CT intensity transformation has a significant impact on CT-MR multimodal registration.

The first row shows the original CT image and corresponding histogram. The second row presents the preprocessed CT image in intensity-transformed scale and its related histogram.

#### Multimodal registration

Registration of CT neonatal head images to the MR neonatal template is a challenging problem not only due its inter-modality nature, but also due to specificities related to neonatal head anatomy and imaging issues. In neonatal head CT/MR images, extracranial tissues are highly variable due to the very fine soft tissues of the scalp, skull discontinuities and fontanels-sutures. In contrast, intracranial soft tissues are physically more constrained and consequently less variable in terms of morphology and appearance. The above considerations led us to choose a two-step intensity-based approach for registration of CT images to the MR neonatal template, as illustrated in the multimodal registration part (dashed box) of Fig 1. At the first step of multimodal registration, the use of intracranial image intensity is expected to provide better results than the use of intensity information from the whole head. This strategy eliminates the effect of extracranial variations in multimodal registration. In the second step, the extracranial parts of the head are used for affine registration between the whole CT image and the MR template. Due to the unavailability of the GRAMFC_T_39-42_ MR intracranial template, this template had to be constructed in the framework of this research. All required steps of co-registration and segmentation, as shown in Fig 1, will be described with more details in the following subsections.

**Fig 3:**
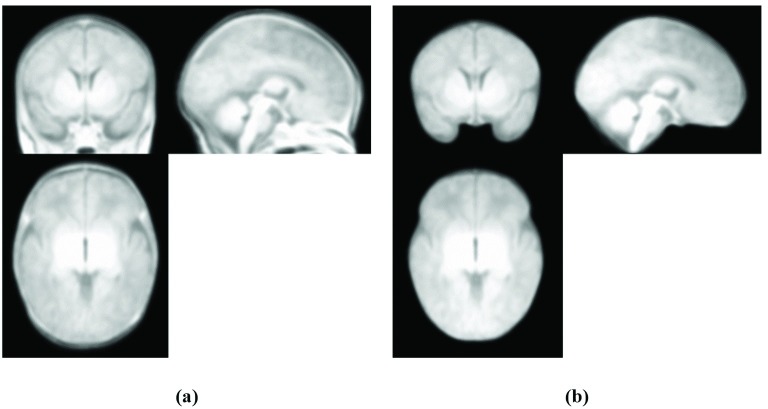
GRAMFC_T_39-42_ template and the proposed intracranial template. (a) GRAMFC_T_39-42_ template [8], (b) Proposed MR intracranial template.

MR Intracranial Template Creation: The same images and the same GRAMFC_T_39-42_ template building procedure described in [8] were used to construct the MR intracranial template. After aligning all MR images to the same stereotaxic space, brain and CSF were extracted from each MR image by the method proposed by Ashburner and Friston [51] using SPM8 software in conjunction with neonatal brain and CSF probabilistic models [19]. The extracted tissues were then binarized by histogram thresholding using the method described by Otsu [48] and were then added and integrated as a brain-CSF mask by applying various morphology operators such as “or”, “closing” and “filling” functions. Intracranial can be extracted by detecting outer surface of dura mater [52, 53]. As no automatic tools for extracting dura mater from neonatal T1 images are available, an expert radiologist performed manual correction on the extracted brain and CSF to add dura mater. After manual correction and revision of the surfaces by the expert and after obtaining the intracranial mask in all images, the MR intracranial images were obtained, averaged and smoothed with a 2 mm Full Width at Half Maximum (FWHM) Gaussian kernel as used in [8, 10] to construct the MR intracranial template. Fig 3 shows the GRAMFC_T_39-42_ template and our proposed intracranial template.

**Fig 4:**
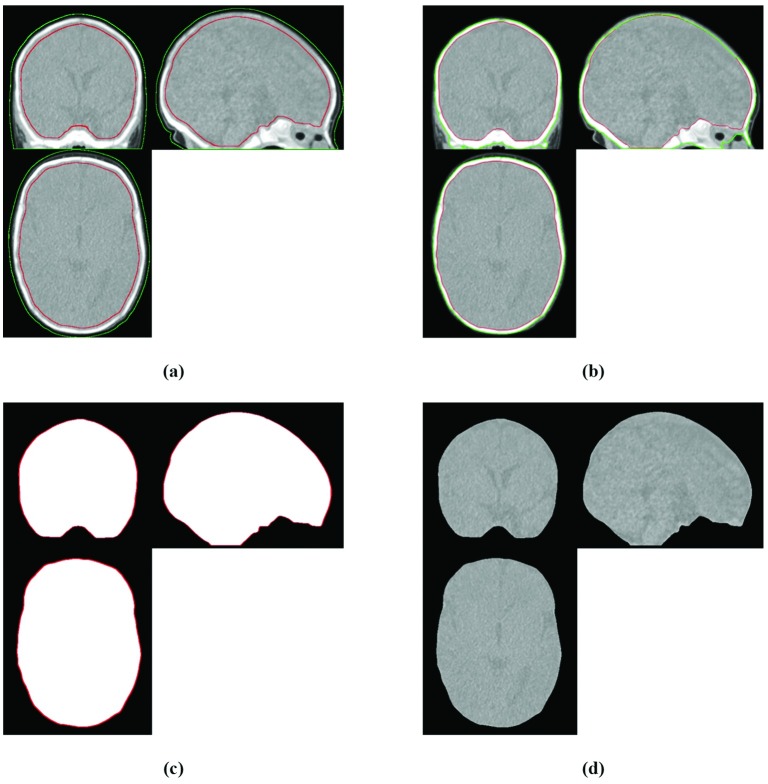
CT intracranial extraction using coupled level sets. (a) The initial coupled level sets. (b) The extracted inner and outer surfaces of cranial bones and fontanels-sutures using the method described in [46]. (c) Corresponding mask of the resulting interior contour. (c) The extracted intracranial CT image obtained by multiplying the mask to the preprocessed CT image. The interior and exterior contours are represented by the red and green colour, respectively.

Extraction of Intracranial CT Data: The coupled level set approach for skull segmentation and fontanel reconstruction in neonatal CT scans was used to extract intracranial CT data [46]. This approach uses hard tissue contrast in CT images, prior information concerning head shape integrated in level set initialization, and a predefined constraint to impose surface reconstruction properties. It applies a pair of interior/exterior surfaces as geodesic active regions propagating towards and interacting with each other. Level set evolution is stopped when they touch each other or encounter the outer (convex) and inner (concave) surfaces of cranial bones using edge information. In the coupled level set method, each moving surface (e.g. interior surface) is influenced not only by its own internal and external forces, but also by a mutual term which is controlled by another (exterior) moving surface. In locations corresponding to fontanels-sutures, these moving surfaces touch each other without crossing over. The resulting contour (inner surface of cranial bones and fontanels-sutures) is assumed to be the intracranial outer surface (Fig 4B). After expert revision of the surface, a mask of the surface was created by the filling operator (Fig 4C) and was used to extract the intracranial CT image (Fig 4D).

**Fig 5:**
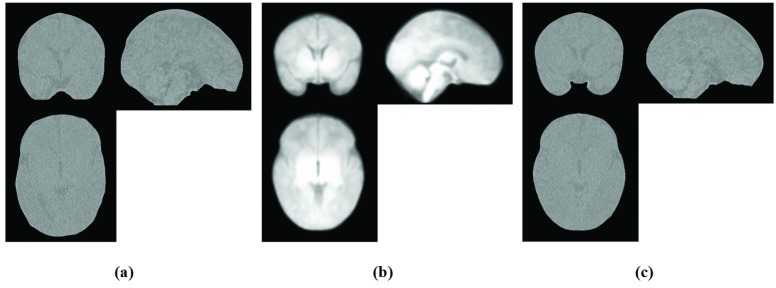
CT-MR intracranial co-registration, first step: Intracranial intensity-based registration. (a) The input moving CT intracranial space. (b) The reference MR intracranial space. (c) The CT intracranial registered to GRAMFC_T_39-42_ intracranial space using affine and non-linear SyN approach [54].

CT-MR Intracranial Co-registration: Intracranial registration consists of two steps. The first step consists of a 12-parameter affine transformation that is used for translation and linear deformation (rotation, scaling and shearing) of the input images to the stereotaxic space as defined by the constructed MR intracranial template. In the second step, a nonlinear deformation with symmetric normalization transformation (SyN) [54] is used to regionally match the subject to the template. The SyN method was shown to have the best performance among 14 different nonlinear registration methods in a comparative study by Klein [55]. It uses symmetric parameterization for geodesic connection of two neuroimages (*I* and *J*) in the diffeomorphic space and is invertible in the discrete domain [54]. Regardless of whether it is fixed or moving, both images are deformed in time along the geodesic path. Fig 5 presents the moving intracranial CT image and the GRAMFC_T_39-42_ intracranial space as a fixed image. The registered intracranial CT data shown in Fig 5C was achieved by applying both affine and non-linear SyN transformations.

**Fig 6:**
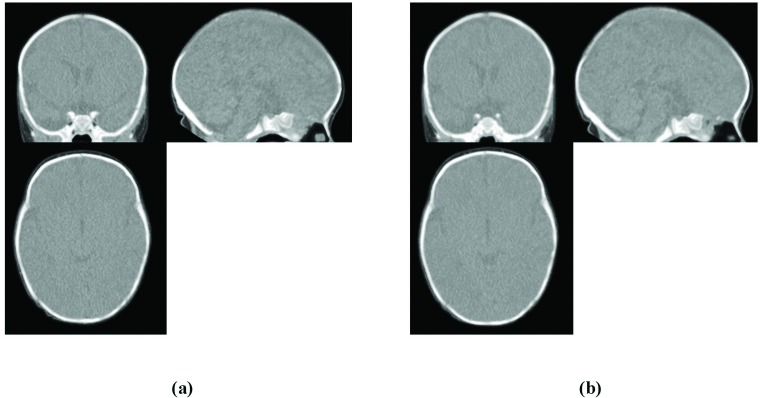
CT'-MR co-registration, second step: whole head intensity-based registration. (a) CT' image obtained by applying the resulting deformation field from CT-MR intracranial co-registration sub-section to the preprocessed CT image. (b) CT'' image achieved by linearly registering CT' to GRAMFC_T_39-42_.

CT'-MR Co-registration: After registering the CT intracranial to the MR intracranial template, the resulting deformation field, which is similar to the elastic model, was applied to the preprocessed CT data to warp the image based on intracranial information and to provide CT' image (Fig 6A). The CT' image was then registered to the MR template by affine transformation using mutual information (MI) similarity measure (Fig 6B). All transformations were applied using the open source toolkit Advanced Normalization Tools (ANTs) [56] built on an Insight ToolKit (ITK) framework [57]. The outputs of this stage (CT'') are CT images that are almost fully aligned with each other and with the MR template. These images are the input of the last part of the proposed method to create the CT template.

#### Template building

The major challenge when constructing a template is to eliminate the bias of the constructed template with respect to the specific subject(s). Groupwise registration is one of the powerful methods used for template building. This method not only incorporates all image information in the registration process (by simultaneously registering all images to the mean of the population), but also eliminates the bias related to a specific reference image in data analysis and computational anatomy. The groupwise registration method proposed by Avants et al. [58] has been integrated into the template construction function by ANTs software. It provides an optimal unbiased template with respect to the input data by using a symmetric diffeomorphic approach and by proposing an unbiased optimization strategy that accounts for appearance and shape variation. This symmetric groupwise normalization (SyGN) method applies SyN energy terms to measure image similarity and minimize diffeomorphism lengths. SyGN has been shown to be an effective tool for template building [58].

To construct the template, a script named “antsMultivariateTemplateConstruction.sh” using cross-correlations [59] as a similarity measure was run on the CT'' images. The number of iterations was set to 4 by default, however as the images were in good alignment with each other, setting the iteration limit to 2 was sufficient for the convergence and creation of a reliable template. Our experiments with different number of iterations from 2 to 5 revealed that the created templates are similar in terms of mean and standard deviation of the MI similarity index. To maintain the connection between CT data and the MR template, the MR template was chosen as the initial template guess. Firstly, the diffeomorphisms between the fixed MR template and individual input images are optimized by minimizing the computed total length between the two. The template appearance with fixed shape and the mappings resulting from the previous step are then optimized by maximizing the similarity of intensity pattern between the template and the set of currently deformed CT images using cross-correlation measure. Finally, by computing a small diffeomorphism that maps the image toward the average shape, the template shape is updated. The above procedure is then reiterated until convergence.

#### Validation

Mutual information (MI) is one of the best similarity measures to compare co-registered inter-modality images. It was used to evaluate the similarity between MR and CT templates. The mutual information *I*(*X*, *Y*) between image *X* (MR template) and image *Y* (CT template) is given by [60]:

$$I(X,Y)=\sum_{x}\sum_{y}p(x,y)\log\frac{p(x,y)}{p(x).p(y)}$$ (1)

where *p*(*x*,*y*) is the joint probability distribution of the two MR and CT templates, and *p*(*x*) and *p*(*y*) are the marginal probability distribution functions of MR and CT templates, respectively. As derived from (5), the MI value is zero when the images share nothing in common and is equal to the entropy of one image when the images are completely registered.

To quantitatively evaluate the constructed neonatal CT template, CT test images were registered to the template in order to determine the similarity between registered images and the template. The affine registration using cross-correlation (CC) measure was initially applied to globally align the test images to the template. The nonlinear SyN registration method and CC measure were then used to locally map the test images to the template. Since CC was used for intra-modality registration to normalize the test images to the CT template, the registered images were evaluated using MI with the proposed CT template, which was smoothed by a Gaussian kernel with 2 mm FWHM as used in [8,10] for fetuses and newborns. For evaluation, two different scenarios were considered. In the first scenario, the leave-one-out cross-validation was applied for 16 images used during template creation and in the second scenario, cross-validation was performed with 10 test CT images.

## Experimental Results and Discussion

### Bimodal MR-CT Head Template Evaluation

**Fig 7:**
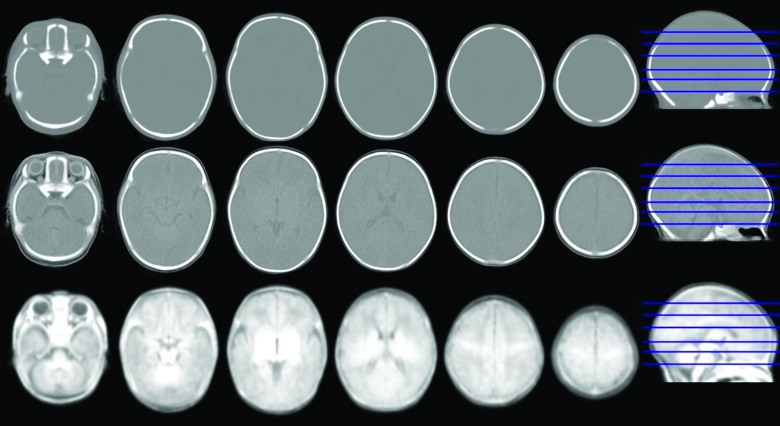
Multi-slice visualization of the proposed neonatal MR-CT head template. The first and second row show some axial slices in Hounsfield units and the intensity-transformed scale of GRAMFC_CT_39-42_, respectively, and the third row shows the corresponding slices from the GRAMFC_T_39-42_ MR template.

Fig 7 shows the constructed bimodal MR-CT template for neonates with 39–42 weeks GA. The new GRAMFC_CT_39-42_ template in Hounsfield units and the intensity rescaled using intensity transformation, as described in the preprocessing subsection, is shown in the first two rows of Fig 7. Multi-slice visualization of the bimodal template qualitatively demonstrates the accuracy of the proposed method and the full alignment of CT and MR templates.

The MI value obtained for the GRAMFC_CT_39-42_ template in Hounsfield units is 1.02 and for the intensity-transformed CT template is 1.17. The resulting value for intensity-transformed CT is greater than the Hounsfield unit CT atlas, which shows that intensity changes of the CT template with an invertible transformation, increases the similarity of these MR and CT templates. Compared to the values of MR template entropy (1.94) and CT template entropy (1.59), the value of 1.17 indicates a good alignment of the two templates.

The similarity of the MR-CT template proposed by Rorden et al. [28] for older adults was computed in order to provide a clearer interpretation of the MI value. The MI value for their high-resolution bimodal MR-CT template was 0.84, when the CT template is expressed in Hounsfield units and 0.88 for the intensity-transformed CT template, which demonstrates that our MR and CT templates are more similar to each other than the template proposed by Rorden et al. and confirms the advantages of the proposed method to create a bimodal neonatal brain template.

### CT Template Evaluation

The CT template was presented in both the intensity-transformed scale and Hounsfield units (Fig 7). To assess the performance of the CT template with the two different intensities and to determine which representation provides better results, all evaluations of image normalization will hereafter be applied to the proposed CT template with both scaling modalities. Table 1 shows the MI values for the two scenarios and for the proposed CT template in Hounsfield units and the intensity-transformed scales. The second and third columns of Table 1 clearly illustrate the out-performance of the proposed CT template using the intensity-transformed scale.

**Table 1:** Mutual information (Mean±SD) between normalized images and neonatal CT templates.

	GRAMFC_CT_39-42_ (intensity-transformed)	GRAMFC_CT_39-42_ (Hounsfield units)	Jafarian et al. CT template (CT_J_)
Leave-one-out	1.23±0.06	0.95±0.06	-
Cross-validation	1.24±0.07	0.98±0.1	0.63±0.07

To compare our template with the only available CT template for newborns proposed by Jafarian et al. [27], 10 test images were first registered to their template using the same registration procedure and parameters as described above. The similarity of the normalized images with the template was then evaluated by MI similarity measure. The average MI values for cross-validation using 10 test images is reported in Table 1. Comparison of the MI values in the first and second columns of Table 1 demonstrates the superiority of the proposed method used for template creation and its capacity to be used as a template. To study the performance of the templates in normalized images, the similarity between normalized images was also examined. For this purpose, forty five different pairs were created from ten test images and each image of each pair was registered to the proposed CT template (GRAMFC_CT_39-42_) and to the CT template proposed by Jafarian et al. (CT_J_) using the same registration parameters. By assuming that *I*_*i*_ and *I*_*j*_ are a pair of images (*I*_*i*_,*I*_*j*_), following registration of these images to the GRAMFC_CT_39-42_ and CT_J_ templates to obtain ${\left(I_{i}^{\prime },I_{j}^{\prime }\right)}_{{GRAMFC\_CT}_{39-42}}$ and ${\left(I_{i}^{\prime },I_{j}^{\prime }\right)}_{{CT}_{J}}$, respectively, cross-correlation was then used to measure the similarity between the two data sets, i.e. ${cc(I_{i}^{\prime },I_{j}^{\prime })}_{{GRAMFC\_CT}_{39-42}}$ and ${cc(I_{i}^{\prime },I_{j}^{\prime })}_{{CT}_{J}}$. The mean and standard deviation of similarity measures of all pairs for each CT template are reported in Table 2, which shows a higher level of similarity between normalized images using our CT template.

**Table 2:** Mean and standard deviation of cross-correlation between 45 image pairs after registration to GRAMFC_CT_39-42_ and CT_J_.

	GRAMFC_CT_39-42_ (intensity-transformed)	GRAMFC_CT_39-42_ (Hounsfield units)	Jafarian et al. CT template (CT_J_)
Cross-Correlation	0.97±0.005	0.87±0.025	0.84±0.023

**Fig 8:**
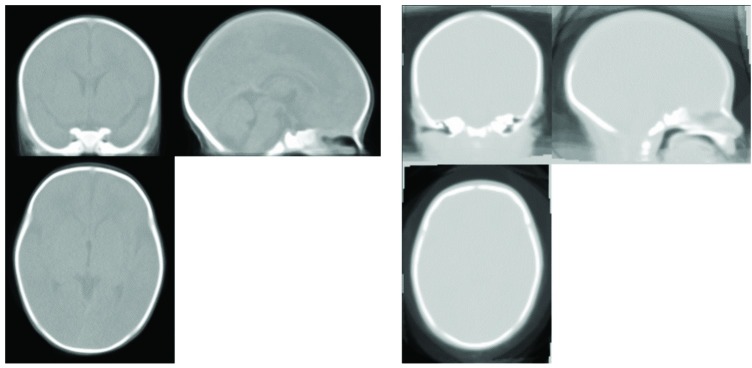
Qualitative comparison of the GRAMFC_CT_39-42_ template (left) and the template proposed by Jafarian et al. [27] (right) for neonates with 39–42 weeks GA.

Fig 8 qualitatively shows the difference between GRAMFC_CT_39-42_ and CT_J_. The template proposed here provides greater anatomic definition compared to CT_J_.

Our bimodal MR-CT neonatal head template was constructed based on the only available and eligible T1 weighted (T1w) MR template which can be considered as a limitation. T1 weighting is mostly used during the preterm and perinatal periods, but the contrast becomes poorer with age until it recovers during the second post-natal year. T2 weighting transitorily enables a better contrast between term and 4–6 months post-term [61]. Another issue is the differing voxel sizes and non-isotropic voxels in the images we used for creating the template. Obviously, high resolution CT images with the same and isotropic voxel sizes are preferred for being used in template construction. However, these images are rarely available in practice due to the difficulties in imaging neonates and high risk imposed by X-ray exposure. Finally, our methodology applied some interpolation/smoothing operations to the images before and during the registration procedures. Although these operations are almost mandatory, further considerations are required to optimize the procedure to improve the accuracy of the created template.

## Conclusion

We present an approach for construction of a bimodal MR-CT head template for newborns with 39–42 weeks GA. Regardless of the neonatal MR template chosen, a two-step intensity-based method was proposed for inter-subject inter-modality registration and a groupwise registration approach was applied to create a CT template. The constructed CT template is the first neonatal template in a standard stereotaxic space similar to MNI which could be used for spatial normalization in CT modality. The CT template was built using intensity-transformed CT data and presented as an intensity-transformed CT template. All evaluations showed that the intensity-transformed CT template is more reliable than the Hounsfield unit template for bimodal MR-CT template and image normalization using popular open source methods. It is noteworthy that the proposed method is not dependent on a specific MR template and can be used to create a CT template in full alignment with any MR template satisfying the previously defined eligibility criteria.

Future directions of this work include: 1) constructing a bimodal MR-CT head atlas consisting of probabilistic maps of cranial bones, fontanels and the scalp in order to enhance the skull and scalp segmentation in MR images, We are developing the methodology of such application and the primary results are being evaluated, 2) application of the created bimodal atlas to neuronal source localization in infants by either high-density electroencephalography or functional optical imaging to demonstrate its superiority versus its monomodal counterparts in terms of localization accuracy.

## Ethical Statement

Institutional Review Board approval was obtained for this study. Ethical permission for this study was provided by the local ethics committee (Commission d’Evaluation Ethique de Recherches Non Interventionelles, CEERNI), CHU Amiens, approval No. 66, 2011. Written informed consent was obtained from each subject’s caregivers.
